# Supplementary material for: Sex-specific Mendelian randomization study of genetically predicted insulin and cardiovascular events in the UK Biobank
Source: Commun Biol. 2019 Sep 5;2:332. doi: 10.1038/s42003-019-0579-z (PMC6728387; doi:10.1038/s42003-019-0579-z)

Supplementary Table 1. Sex-specific associations of each genetic variant for insulin with myocardial infarction, angina and heart failure

SNP	Effect allele	MI in men		MI in women		Angina in men		Angina in women		Heart failure in men		Heart failure in women	
		beta	SE	beta	SE	beta	SE	beta	SE	beta	SE	beta	SE
rs1530559	A	0.01	0.01	-0.02	0.03	0.01	0.01	-0.003	0.02	0.04	0.02	0.02	0.04
rs2745353	T	0.02	0.01	0.04	0.03	0.02	0.01	0.03	0.02	-0.01	0.02	-0.02	0.04
rs2820436	C	0.02	0.01	-0.03	0.03	0.02	0.01	0.001	0.02	0.02	0.02	-0.09	0.04
rs2972143	G	0.06	0.01	-0.01	0.03	0.04	0.01	0.02	0.02	0.03	0.02	-0.06	0.04
rs4865796	A	0.03	0.02	0.01	0.03	0.01	0.01	-0.02	0.02	-0.01	0.02	0.06	0.04
rs731839	G	0.03	0.01	-0.001	0.03	0.03	0.01	0.01	0.02	-0.03	0.02	-0.001	0.04
rs983309	T	-0.002	0.02	-0.01	0.04	-0.01	0.02	-0.04	0.03	-0.03	0.04	0.04	0.05

MI, myocardial infarction; SE, standard error

Supplementary Table 2. Sensitivity analyses on the associations of genetically predicted insulin and insulin adjusted for BMI with myocardial infarction, angina and heart failure using different analysis methods

Outcomes	Sex	Methods	Serum insulin			Serum insulin adjusted for BMI		
			#SNPs	OR	95% CI	#SNPs	OR	95% CI
Myocardial infarction	Overall	WM	7	**2.93**	**1.25 to 6.86**	9	**3.09**	**1.37 to 7.00**
		MR-PRESSO	7	**2.87**	**1.07 to 7.70**	9	**2.63**	**1.21 to 5.70**
		MBE	7	1.65	0.39 to 6.98	9	4.47	0.93 to 21.5
	Men	WM	7	**3.95**	**1.47 to 10.6**	9	**4.82**	**1.79 to 13.0**
		MR-PRESSO	7	**4.27**	**1.26 to 14.5**	9	**3.60**	**1.25 to 10.3**
		MBE	7	4.25	0.88 to 20.5	9	7.25	0.94 to 56.1
	Women	WM	7	0.75	0.14 to 3.88	9	0.86	0.21 to 3.61
		MR-PRESSO	7	0.80	0.20 to 3.28	9	0.96	0.50 to 1.85
		MBE	7	0.68	0.07 to 6.87	9	0.76	0.10 to 5.98
Angina	Overall	WM	7	1.79	0.82 to 3.91	9	**2.67**	**1.29 to 5.51**
		MR-PRESSO	7	2.05	0.71 to 5.97	9	**2.47**	**1.21 to 5.06**
		MBE	7	1.03	0.20 to 5.15	9	**4.25**	**1.22 to 14.7**
	Men	WM	7	**2.58**	**1.06 to 6.27**	9	**4.34**	**1.79 to 10.5**
		MR-PRESSO	7	**2.93**	**1.04 to 8.28**	9	**2.74**	**1.05 to 7.15**
		MBE	7	2.33	0.56 to 9.65	9	**6.92**	**1.52 to 31.4**
	Women	WM	7	0.98	0.30 to 3.17	9	1.40	0.49 to 4.02
		MR-PRESSO	7	1.09	0.29 to 4.15	9	1.00	0.40 to 2.52
		MBE	7	0.54	0.07 to 4.19	9	2.06	0.41 to 10.3
Heart failure	Overall	WM	7	0.70	0.19 to 2.50	9	1.16	0.35 to 3.84
		MR-PRESSO	7	0.98	0.29 to 3.33	9	1.18	0.38 to 3.72
		MBE	7	0.59	0.10 to 3.47	9	1.33	0.23 to 7.70
	Men	WM	7	0.61	0.12 to 2.96	9	1.98	0.47 to 8.34
		MR-PRESSO	7	1.17	0.21 to 6.53	9	1.90	0.51 to 7.10
		MBE	7	0.47	0.04 to 5.46	9	1.79	0.21 to 15.4
	Women	WM	7	1.25	0.11 to 14.8	9	1.22	0.12 to 12.5
		MR-PRESSO	7	0.64	0.03 to 13.1	9	0.78	0.08 to 7.51
		MBE	7	2.45	0.06 to 93.2	9	2.05	0.03 to 124.3

BMI, body mass index; CI, confidence interval; MBE, mode based estimation; OR, odds ratio; SNP, single nucleotide polymorphism; WM, weighted median

Supplementary Table 3. Sensitivity analyses on the associations of genetically predicted insulin and insulin adjusted for BMI with myocardial infarction, angina and heart failure with SNPs related to alcohol intake

**Outcomes**	**Sex**	**Insulin**			**Insulin adjusted for BMI**		
		**#SNPs**	**OR**	**95% CI**	**#SNPs**	**OR**	**95% CI**
Myocardial infarction	Overall	9	**2.61**	**1.43 to 4.80**	11	**2.61**	**1.54 to 4.41**
	Men	9	**3.55**	**1.65 to 7.65**	11	**3.59**	**1.61 to 8.01**
	Women	9	0.97	0.32 to 2.89	11	0.97	0.31 to 3.11
Angina	Overall	9	**2.19**	**1.12 to 4.27**	11	**2.09**	**1.15 to 3.80**
	Men	9	**2.63**	**1.35 to 5.15**	11	**2.60**	**1.33 to 5.08**
	Women	9	1.57	0.63 to 3.94	11	1.42	0.67 to 3.01
Heart failure	Overall	9	0.94	0.41 to 2.20	11	1.15	0.52 to 2.57
	Men	9	1.15	0.41 to 3.21	11	1.77	0.68 to 4.62
	Women	9	0.59	0.09 to 3.80	11	0.40	0.06 to 2.86

BMI, body mass index; CI, confidence interval; OR, odds ratio; SNP, single nucleotide polymorphism

Supplementary Table 4. Sensitivity analyses showing the associations of genetically predicted insulin and insulin adjusted for BMI with myocardial infarction, angina and heart failure including all potentially pleiotropic SNPs

**Outcomes**	**Sex**	**Insulin**			**Insulin adjusted for BMI**		
		**#SNPs**	**OR**	**95% CI**	**#SNPs**	**OR**	**95% CI**
Myocardial infarction	Overall	12	1.28	0.58 to 2.82	12	**2.75**	**1.68 to 4.53**
	Men	12	1.83	0.80 to 4.21	12	**3.59**	**1.67 to 7.71**
	Women	12	0.40	0.14 to 1.14	12	0.99	0.31 to 3.14
Angina	Overall	12	1.41	0.72 to 2.77	12	**1.89**	**1.04 to 3.41**
	Men	12	1.65	0.82 to 3.32	12	2.10	0.98 to 4.50
	Women	12	1.07	0.48 to 2.41	12	1.55	0.75 to 3.17
Heart failure	Overall	12	1.22	0.60 to 2.46	12	1.48	0.60 to 3.67
	Men	12	1.61	0.70 to 3.72	12	2.16	0.86 to 5.44
	Women	12	0.62	0.15 to 2.53	12	0.58	0.08 to 4.24

BMI, body mass index; CI, confidence interval; OR, odds ratio; SNP, single nucleotide polymorphism

Supplementary Table 5. Sensitivity analyses on the associations of genetically predicted BMI-adjusted insulin with myocardial infarction, angina and heart failure in men excluding rs3822072

Outcomes	#SNPs	Odds ratio	95% confidence interval
Myocardial infarction	8	**3.87**	**1.45 to 10.3**
Angina	8	**3.20**	**1.42 to 7.23**
Heart failure	8	2.01	0.58 to 6.96

BMI, body mass index; SNP, single nucleotide polymorphism; BMI, body mass index
Inverse variance weighting with random effect was used.

Supplementary Table 6. Associations of genetically predicted insulin and insulin adjusted for BMI with cardiovascular disease risk factors overall and by sex for figure 1

Outcomes	Sex	Insulin		Insulin adjusted for BMI	
		Beta coefficient	95% confidence interval	Beta coefficient	95% confidence interval
LDL	Overall	0.07	-0.41, 0.55	-0.17	-0.85, 0.52
ApoB	Overall	0.36	-0.53, 1.24	**0.76**	**0.21, 1.30**
SBP	Overall	**0.36**	**0.10, 0.62**	**0.48**	**0.37, 0.59**
	Men	0.29	-0.01, 0.58	**0.55**	**0.34, 0.75**
	Women	**0.43**	**0.11, 0.75**	**0.45**	**0.22, 0.67**
DBP	Overall	0.19	-0.12, 0.51	0.15	-0.10, 0.41
	Men	0.04	-0.29, 0.37	0.14	-0.10, 0.38
	Women	0.34	-0.02, 0.69	0.17	-0.20, 0.54
Reticulocyte	Overall	**0.69**	**0.40, 0.97**	**0.69**	**0.51, 0.87**
count	Men	**0.81**	**0.45, 1.17**	**0.82**	**0.56, 1.08**
	Women	**0.61**	**0.26, 0.96**	**0.60**	**0.35, 0.84**

ApoB, apolipoprotein; BMI, body mass index; DBP, diastolic blood pressure; LDL, low density lipoprotein; SBP, systolic blood pressure.
Inverse variance weighting with random effect was used for the associations of insulin and BMI-adjusted insulin with SBP, DBP and reticulocyte count, of insulin with ApoB, and of BMI-adjusted insulin with LDL; MR-PRESSO with correction for outliers was used for the association of insulin with LDL, and of BMI-adjusted insulin with ApoB.

Supplementary Figure 1. Scatter plot for genetically predicted insulin and myocardial infarction, angina and heart failure

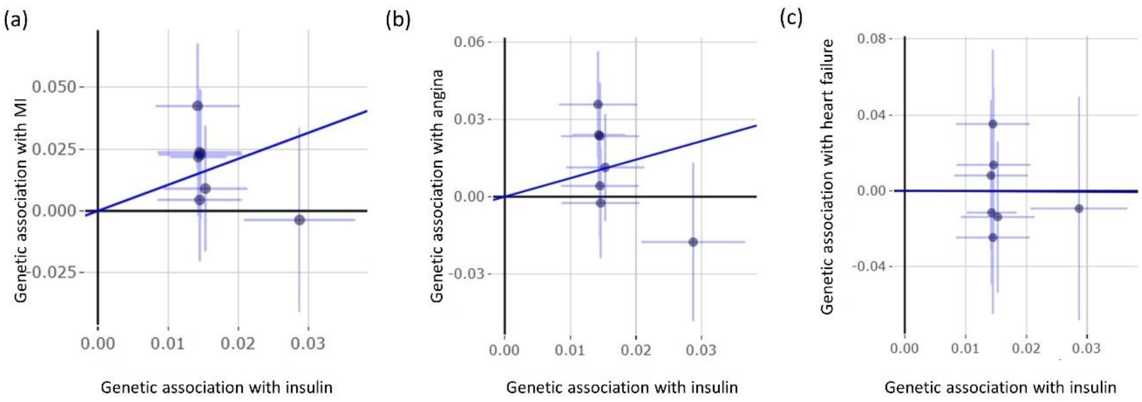

Supplement: Supplementary file 1 — Supplementary Information [file 42003_2019_579_MOESM1_ESM.pdf]
