# Supplementary material for: Sex-specific Mendelian randomization study of genetically predicted insulin and cardiovascular events in the UK Biobank
Source: Commun Biol. 2019 Sep 5;2:332. doi: 10.1038/s42003-019-0579-z (PMC6728387; doi:10.1038/s42003-019-0579-z)
Supplement: Supplementary file 2 — Reporting Summary [file 42003_2019_579_MOESM2_ESM.pdf]

nature research

Corresponding author(s): C Mary Schooling

Last updated by author(s): Jul 30, 2019

Reporting Summary

Nature Research wishes to improve the reproducibility of the work that we publish. This form provides structure for consistency and transparency in reporting. For further information on Nature Research policies, see Authors & Referees and the Editorial Policy Checklist .

Statistics

For all statistical analyses, confirm that the following items are present in the figure legend, table legend, main text, or Methods section.

n/a	Confirmed	
☐	☒	The exact sample size (n) for each experimental group/condition, given as a discrete number and unit of measurement
☐	☒	A statement on whether measurements were taken from distinct samples or whether the same sample was measured repeatedly
☐	☒	The statistical test(s) used AND whether they are one- or two-sided<br>*Only common tests should be described solely by name; describe more complex techniques in the Methods section.*
☐	☒	A description of all covariates tested
☐	☒	A description of any assumptions or corrections, such as tests of normality and adjustment for multiple comparisons
☐	☒	A full description of the statistical parameters including central tendency (e.g. means) or other basic estimates (e.g. regression coefficient) AND variation (e.g. standard deviation) or associated estimates of uncertainty (e.g. confidence intervals)
☒	☐	For null hypothesis testing, the test statistic (e.g. F, t, r) with confidence intervals, effect sizes, degrees of freedom and P value noted<br>*Give P values as exact values whenever suitable.*
☒	☐	For Bayesian analysis, information on the choice of priors and Markov chain Monte Carlo settings
☒	☐	For hierarchical and complex designs, identification of the appropriate level for tests and full reporting of outcomes
☐	☒	Estimates of effect sizes (e.g. Cohen's d, Pearson's r), indicating how they were calculated

Our web collection on statistics for biologists contains articles on many of the points above.

Software and code

Policy information about availability of computer code

Data collection	"ukbmd5", "ukbunpack", "ukbconv" and "ukbgene" were used for data validation, unpacking, format conversion and obtaining genetic data. They are available for the approved UK Biobank application (#42468), in the online system (https://bbams.ndph.ox.ac.uk/ams/).
Data analysis	All statistical analyses were conducted using R version 3.4.4 (R Foundation for Statistical Computing, Vienna, Austria) and the R package “MendelianRandomization” (Yavorska, O & Burgess S, Int J Epidemiol, 2017; 46: 1734-9).

For manuscripts utilizing custom algorithms or software that are central to the research but not yet described in published literature, software must be made available to editors/reviewers. We strongly encourage code deposition in a community repository (e.g. GitHub). See the Nature Research guidelines for submitting code & software for further information.

Data

Policy information about availability of data

All manuscripts must include a data availability statement. This statement should provide the following information, where applicable:
- Accession codes, unique identifiers, or web links for publicly available datasets
- A list of figures that have associated raw data
- A description of any restrictions on data availability

The main outcomes are from the UK Biobank under application (#42468). The data is available from the UK Biobank upon request. Data on coronary artery disease/ myocardial infarction have also been contributed by CARDIoGRAMplusC4D investigators and have been downloaded from www.CARDIOGRAMPLUSC4D.ORG. Genetic associations with lipids were obtained from the Global Lipids Genetics Consortium Results, downloaded from http://csg.sph.umich.edu//abecasis/public/ lipids2013/. Genetic associations with apolipoprotein B were obtained from the GWAS of Kettunen et al. (2016), downloaded from http:// www.computationalmedicine.fi/data#NMR_GWAS. Genetic associations with blood pressure and reticulocyte count were from the UK biobank GWAS results, downloaded from http://www.nealelab.is/uk-biobank/, the results of the GWAS and heritability analyses conducted by Neale Lab. The summary data are publicly available.

Field-specific reporting

Please select the one below that is the best fit for your research. If you are not sure, read the appropriate sections before making your selection.

- [x] Life sciences
- [ ] Behavioural & social sciences
- [ ] Ecological, evolutionary & environmental sciences

For a reference copy of the document with all sections, see nature.com/documents/nr-reporting-summary-flat.pdf

Life sciences study design

All studies must disclose on these points even when the disclosure is negative.

Sample size	Mendelian randomization requires large sample size (Burgess S, Int J Epidemiol, 2014; 43: 922-9), so we used by far the largest available genome-wide association studies (GWAS) to obtain the genetic predictors for insulin and insulin resistance (n=108,557), and the genetic associations with the outcomes. Specifically, the associations with myocardial infarction (MI), angina and heart failure were obtained in 392,010 white British from the UK Biobank to assess the sex-specific role of genetically predicted insulin and insulin resistance in MI (14,442 cases, 77% men), angina (21,939 cases, 65% men), heart failure (5,537 cases, 71% men). We also obtained the genetic associations with low density lipoprotein (LDL) cholesterol (188,577 participants of European descent and 7,898 participants of non-European descent), apolipoprotein B (n=24,925), blood pressure and reticulocyte count (n=361,194).
Data exclusions	To control for population stratification, we restricted our analysis to participants with self-reported and genetically validated white British ancestry. For quality control, we also excluded participants with 1) excess relatedness (more than 10 putative third-degree relatives) or 2) mismatched information on sex between genotyping and self-report, or 3) sex-chromosomes not XX or XY, or 4) poor-quality genotyping based on heterozygosity and missing rates>1.5%.
Replication	We replicated the analysis for MI in CARDIoGRAMplusC4D 1000 Genomes, another large genetic study (ischemic heart disease (IHD) cases n=60,801, others=123,504), with ~70% of the cases MI.
Randomization	Mendelian randomization takes advantage of genetic endowment randomly allocated at conception, to obtain unconfounded estimates.
Blinding	The investigators were blinded to the genotyping information during data collection.

Reporting for specific materials, systems and methods

We require information from authors about some types of materials, experimental systems and methods used in many studies. Here, indicate whether each material, system or method listed is relevant to your study. If you are not sure if a list item applies to your research, read the appropriate section before selecting a response.

Materials & experimental systems

n/a	Involved in the study	
x		Antibodies
x		Eukaryotic cell lines
x		Palaeontology
x		Animals and other organisms
	x	Human research participants
x		Clinical data

Methods

n/a	Involved in the study	
x		ChIP-seq
x		Flow cytometry
x		MRI-based neuroimaging

Human research participants

Policy information about studies involving human research participants

Population characteristics	Genetic associations with insulin and insulin resistance were taken from a large meta-analysis of GWAS, conducted in adults (n=108,557; mean age, 50.6 years; ~53% men) of European ancestry, without diabetes. Genetic associations with MI, angina and heart failure were obtained using individual data in the UK Biobank (under the application #42468), with validation for MI using CARDIoGRAPMplusC4D 1000 Genomes. The UK Biobank recruited 502,713 individuals aged 40-69 years, mean age 56.5 years. After quality control, we identified 392,010 white British in the UK Biobank, with 14,442 cases of MI (77% men), 21,939 cases of angina (65% men), and 5,537 cases of heart failure (71% men). CARDIoGRAMplusC4D 1000 Genomes is a large genetic study (IHD cases n=60,801, others=123,504), with ~70% of the cases MI. The participants are largely of European descent (77%) with detailed phenotyping based on medical records, clinical diagnosis, as well as medications, or indicative symptoms or procedures, such as revascularization, and/or angiographic evidence of stenosis. Genetic associations with LDL-cholesterol (as inverse normal transformed effect sizes) were obtained from the Global Lipids Genetics Consortium Results including 188,577 participants of European descent and 7,898 participants of non-European descent, mean age 55.2 years. Genetic associations with ApoB (as inverse normal transformed effect sizes) were obtained from a meta-analysis GWAS of metabolomics in 24,925 Europeans (45% men). We obtained overall and sex-specific genetic associations with blood pressure and reticulocyte count from the UK Biobank summary statistics, provided by Neale Lab (http://www.nealelab.is/uk-biobank/), in 361,194 white British (194,174, 46% men).
Recruitment	The UK Biobank is an ongoing large prospective cohort study. The UK Biobank recruited 502,713 individuals aged 40-69 years, mean age 56.5 years, from England, Scotland and Wales between 2006 and 2010, 94% self-reported European ancestry, 45.6% men, median follow-up time currently 11.1 years.
Ethics oversight	The UK Biobank has already received ethical approval from the Research Ethics Committee and participants provided written informed consent. The analysis of other publicly available data or summary statistics does not require additional ethical approval.

Note that full information on the approval of the study protocol must also be provided in the manuscript.
